# Machine Learning-Based Classification of Dynamic Damage Modes in Rock–Concrete Composites Under Impact Loading

**DOI:** 10.3390/ma19214624

**Published:** 2026-10-30

**Authors:** Zhixuan Gao, Quanmin Xie, Yuzhu Guo, Yuntian Wang, Jin Wu

**Affiliations:** 1 Hubei Key Laboratory of Blasting Engineering, Jianghan University, Wuhan 430056, China; 2 School of Civil and Environmental Engineering, Changsha University of Science & Technology, Changsha 410114, China; 3 School of Urban Construction, Chengdu Polytechnic, Chengdu 610041, China; 4 Sichuan Provincial Engineering Research Center of Resource Utilization for Agricultural and Forestry Wastes, Chengdu 611130, China; 5 College of Civil Engineering and Architecture, Zhejiang University, Hangzhou 310058, China

**Keywords:** rock–concrete composites, dynamic impact, acoustic emission, damage classification, machine learning

## Abstract

In tunnel engineering, rock–concrete composite structures may be subjected to dynamic loading induced by earthquakes, blasting, and rockbursts. This study investigated the dynamic response and damage evolution of granite- and sandstone-based composites with ordinary or fiber-reinforced concrete using split Hopkinson pressure bar (SHPB) impact tests at different impact velocities. Acoustic emission (AE) monitoring was combined with a Gaussian mixture model–support vector machine (GMM–SVM) framework to classify damage modes based on the rise-time-to-peak-amplitude ratio (RA) and average frequency (AF). Across the four specimen types, dynamic compressive strength ranged from 37.35 to 57.65 MPa under low-impact conditions and from 102.73 to 120.75 MPa under ultra-high-impact conditions. The corresponding dissipated energy values ranged from 34.19 to 64.12 J and from 192.98 to 266.81 J, respectively. Granite-based specimens exhibited a more pronounced double-peak response, whereas sandstone-based specimens showed more continuous post-peak softening. Fiber reinforcement improved post-peak load-bearing stability, although its effect on energy dissipation varied with impact velocity. AE activity was concentrated mainly during the early impact stage. Under medium-impact-velocity conditions, where damage-mode classification was performed, shear damage dominated in granite-based specimens (56.0% for GC-1 and 53.4% for GCF-1), whereas tensile damage dominated in sandstone-based specimens (52.6% for SC-1 and 59.9% for SCF-1). Fiber reinforcement increased the tensile-damage ratio in granite-based specimens from 44.0% to 46.6%. These findings clarify how rock type and fiber reinforcement influence the dynamic response and damage evolution of rock–concrete composites.

## 1. Introduction

With the continued growth in demand for transportation infrastructure, the scale of tunnel construction has expanded steadily [1,2], and an increasing number of mountain tunnels are being constructed at greater depths. Rock–concrete composites have been widely employed in large-scale engineering structures, including tunnels [3,4,5]. As a natural material, rock generally exhibits a relatively dense internal structure, whereas concrete, as an artificial material, contains randomly distributed initial pores and cracks resulting from the presence and interaction of aggregates and mortar. Consequently, the two materials exhibit substantial differences in their internal microstructural characteristics. Recent studies on heterogeneous engineering materials have also demonstrated that internal microstructural characteristics and phase distributions can significantly influence mechanical responses and damage evolution [6]. The rock–concrete interfacial transition zone (ITZ) is mechanically vulnerable because the two materials have dissimilar mechanical and microstructural properties. This mismatch can induce local stress concentrations, affect stress transfer, and promote damage initiation and propagation [7,8]. Under external loading, concrete support structures bonded to rock surfaces in tunnels may experience damage in this region, potentially compromising structural safety.

Considerable research has been conducted on rock–concrete interfaces, with increasing attention being paid to their mechanical properties, including compressive strength [9], tensile strength [10], and shear strength [11,12], as well as to their implications for durability [13] and numerical simulation [14]. Previous studies have demonstrated that interface roughness, fracture behavior, and energy dissipation play key roles in influencing the fracture characteristics of rock–concrete interfaces [15,16,17].

Most existing studies have focused on quasi-static tests at relatively low strain rates. In tunnel engineering, however, rock–concrete structures are often subjected to dynamic loading caused by earthquakes, rockbursts, blasting, and explosive impacts. Under these conditions, the mechanical response of rock–concrete composites differs markedly from that under static loading and exhibits a more pronounced strain-rate effect [18,19]. A clear understanding of the dynamic mechanical behavior of rock–concrete composite structures is essential for the design and safety assessment of protective structures in underground engineering. In recent years, several studies have investigated how loading rate and interface characteristics affect the dynamic mechanical behavior of rock–concrete composites [20,21,22,23]. In parallel, machine learning and data-driven methods have increasingly been applied to damage assessment in engineering materials, including acoustic emission (AE)-based characterization of damage progression and failure modes in concrete structures, as well as data-driven modeling of damage evolution and failure prediction in composite materials [24,25,26,27]. However, the internal damage evolution and dynamic failure modes of rock–concrete composites incorporating different rock types remain insufficiently understood. In addition, the effects of rock type and fiber reinforcement on the dynamic load-bearing behavior, energy dissipation characteristics, and damage evolution of rock–concrete composite systems remain insufficiently clarified. Therefore, dynamic impact tests of rock–concrete composite specimens incorporating different rock types and concrete reinforcement conditions are needed to further investigate the factors governing their dynamic response.

Acoustic emission (AE) technology is an effective nondestructive testing technique for dynamically monitoring material damage evolution through the detection of elastic waves released during crack initiation, propagation, and fracture. It has therefore been widely used to assess damage in brittle materials, such as rock and concrete [28,29,30,31]. Characteristic parameters of AE signals, including hit count, ring count, duration, amplitude, and energy, can reflect crack activity within materials and provide valuable information for characterizing damage evolution and failure mechanisms. In recent years, extensive research has been conducted worldwide using AE technology for material damage identification, yielding important findings on crack propagation, fracture processes, and damage assessment [32]. These studies further demonstrate the potential of AE technology for monitoring damage in complex materials.

However, existing AE studies have largely focused on quasi-static loading conditions, with relatively limited attention being devoted to the internal damage evolution of rock–concrete composite structures under dynamic impact loading. Unlike static loading, failure under dynamic impact is highly transient, with crack initiation, propagation, and coalescence occurring over a very short time interval. As a result, AE signals under dynamic loading tend to exhibit intense high-frequency bursts and short durations, making individual signals more difficult to distinguish. In addition, the intense stress waves generated during loading may compromise AE sensor stability and signal acquisition quality, thereby limiting the application of AE monitoring under dynamic conditions. Therefore, further investigation is needed to characterize the internal damage evolution and damage modes of rock–concrete composite specimens subjected to high-strain-rate impact loading.

Based on the above research gaps, the mechanical response and damage evolution of rock–concrete composite structures under dynamic impact loading were investigated for different rock types and fiber-reinforcement conditions. A split Hopkinson pressure bar (SHPB) test system was employed to perform dynamic compression tests on four types of composite specimens, namely, granite–concrete, granite–fiber-reinforced concrete, sandstone–concrete, and sandstone–fiber-reinforced concrete, under different impact velocities. The mechanical responses were analyzed to elucidate the effects of impact velocity, rock type, and fiber reinforcement on the dynamic load-bearing behavior of the composite specimens. In addition, AE monitoring was conducted to characterize damage evolution during dynamic impact, and a Gaussian mixture model–support vector machine (GMM–SVM) method was employed for damage classification based on AE RA-AF parameters. The tensile and shear damage modes of different rock–concrete composite systems were quantitatively identified, providing further insight into their failure mechanisms and internal damage evolution under dynamic impact.

## 2. Experimental Program

### 2.1. Specimen Design

Granite and sandstone were selected as the rock materials and combined separately with ordinary or fiber-reinforced concrete to produce rock–concrete composite specimens. The granite and sandstone were processed separately into individual rock blocks and were not combined with each other in a single block. For each rock type, the rock block was prepared independently for the fabrication of a corresponding rock–concrete composite plate. Before casting, the contact surface between the rock and concrete was pretreated to enhance interfacial bonding. The treated rock block was then placed in a mold, and ordinary or fiber-reinforced concrete was cast against the rock surface to form an integral composite plate. Thus, granite–concrete and sandstone–concrete composite plates were fabricated separately, with each plate containing only one type of rock bonded to the concrete.

Two types of concrete were used, namely, ordinary concrete and fiber-reinforced concrete. P.O. 42.5 ordinary Portland cement, classified according to the Chinese national standard GB 175-2023 (Common Portland Cement), was used as the binder. For international reference, ASTM C150/C150M-24 (Standard Specification for Portland Cement) defines Type I Portland cement as intended for general use when the special properties specified for other cement types are not required. Since the cement used in this study was classified according to GB 175-2023, the ASTM Type I classification is provided for reference only, and no direct equivalence between the two classifications is implied. Natural river sand with a fineness modulus of 2.3 was used as the fine aggregate, whereas crushed limestone with a particle size of 5–10 mm was used as the coarse aggregate. A high-performance polycarboxylate-based superplasticizer was added to enhance concrete workability. Polypropylene synthetic steel-like fibers were incorporated into the fiber-reinforced concrete at a dosage of 10 kg/m^3^. This dosage was adopted as a fixed experimental parameter to investigate the influence of fiber bridging on crack propagation and dynamic damage evolution under impact loading, rather than to determine the optimum fiber content. The selected dosage falls within the application-dependent range reported for concrete macrofibers. Youm and Moon [33] noted that macrofiber dosages can reach 10 kg/m^3^ depending on the application and experimentally investigated the impact behavior of polypropylene macrofiber-reinforced concrete. The mix proportions of ordinary concrete and fiber-reinforced concrete are presented in Table 1. The basic properties of the rock and concrete materials used in this study are summarized in Table 2.

After casting, the rock–concrete composite plates were subjected to standard curing for 28 d. The cylindrical specimens were subsequently obtained by core drilling the cured composite plates. Core drilling was performed perpendicular to the rock–concrete contact surface so that each extracted core contained both the rock and concrete sections, together with the intervening interface. The cores were then cut to the required dimensions, yielding cylindrical composite specimens with a diameter of 75 mm and an overall height of 64 mm.

**Figure 1:**
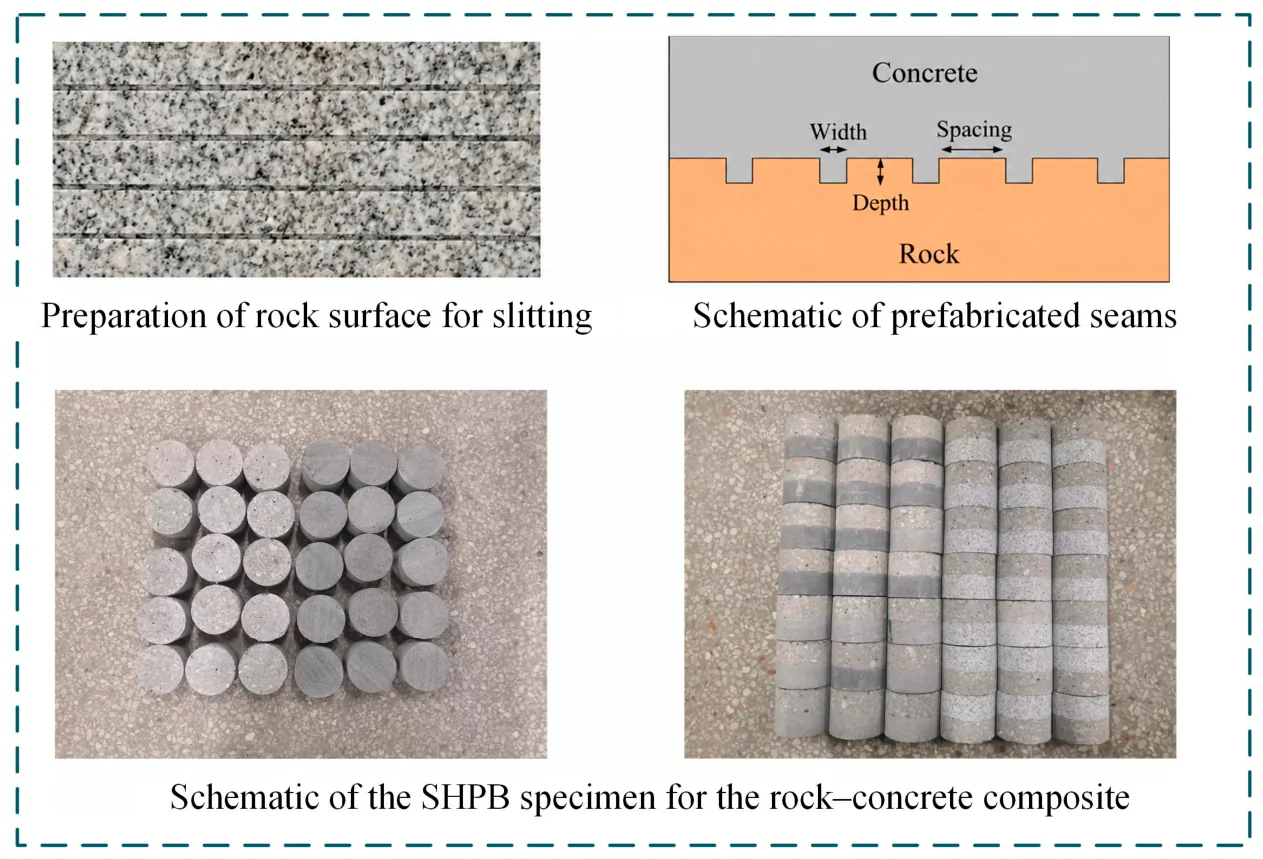
Schematic of specimen preparation.

The specimen dimensions were selected primarily with consideration of the dynamic stress equilibrium requirements of SHPB testing. The specimen diameter of 75 mm matched the diameter of the pressure bars, while the overall height of 64 mm resulted in a height-to-diameter ratio (H/D) of approximately 0.85. The relatively short specimen length was adopted to reduce the stress-wave transit time and facilitate the establishment of dynamic stress equilibrium through repeated wave reflections during impact loading. Meanwhile, the rock and concrete sections were each 32 mm high, placing the rock–concrete interface at the mid-height of the specimen and ensuring equal axial dimensions for the two constituent materials. The specimen preparation procedure is schematically illustrated in Figure 1.

Four types of composite specimens were classified according to the combinations of rock and concrete types: granite–ordinary concrete (GC), granite–fiber-reinforced concrete (GCF), sandstone–ordinary concrete (SC), and sandstone–fiber-reinforced concrete (SCF).

### 2.2. Test Setup

Dynamic impact tests of the rock–concrete composites were performed using an SHPB test system. The SHPB system mainly comprises an incident bar, a transmission bar, and an absorption bar. The pressure bars were made of elastic steel with a density of 7850 kg/m^3^, a Young’s modulus of 210 GPa, and a Poisson’s ratio of 0.30. The longitudinal wave velocity and bar diameter were 5172.2 m/s and 75 mm, respectively. During the test, a pneumatic launcher was used to drive the projectile into the incident bar, generating the incident stress wave ε_i_. The incident wave then propagated along the incident bar to the loading end of the specimen. Owing to the impedance mismatch between the pressure bar and the specimen, part of the stress wave was reflected as ε_r_, whereas the remainder propagated through the specimen and entered the transmission bar as ε_t_.

**Figure 2:**
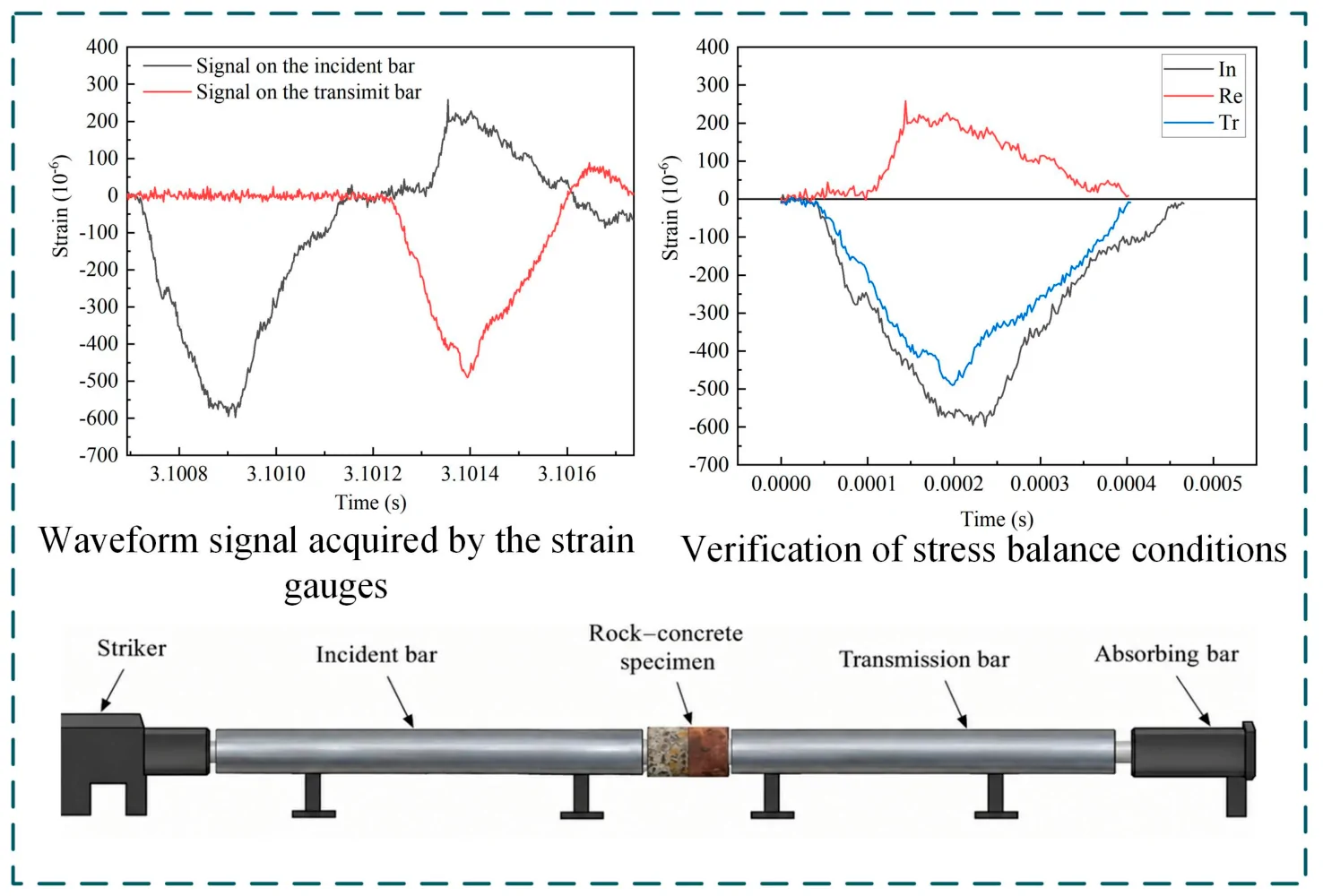
Schematic of the SHPB dynamic impact test.

Different impact loading conditions were obtained by adjusting the pneumatic pressure. Four impact velocity levels, namely, low, medium, high, and ultra-high, were used by varying the applied pneumatic pressure. The dynamic mechanical response and damage evolution of the rock–concrete composites were then investigated under different impact velocities. During each test, the incident, reflected, and transmitted strain-wave signals were recorded using a dynamic strain data acquisition system. The dynamic stress, strain, and strain rate of the specimens were derived from the recorded wave signals. The specimens were positioned between the incident and transmission bars. Before testing, the pressure bar system was carefully aligned to ensure that the axes of all bars were coincident. The validity of the tests was also verified based on the stress-equilibrium condition. The recorded strain-wave data and a schematic illustration of the SHPB dynamic impact test setup are shown in Figure 2.

To minimize the effect of contact friction between the specimen end faces and the pressure bars on the stress state of the specimens, a small amount of Vaseline was applied to the contact interfaces between the specimen and the incident and transmission bars. A copper pulse shaper with a diameter of 20 mm and a thickness of 1 mm was placed at the loading end to increase the rise time of the incident stress wave and facilitate stress equilibrium within the specimen. Three replicate tests were conducted under each impact-velocity condition to assess the repeatability of the experimental results, and a representative test was selected for detailed quantitative analysis.

AE monitoring was performed using a Sensor Highway III AE data acquisition system (MISTRAS, Princeton, NJ, USA) equipped with PK6I AE sensors. During the tests, the AE sensors were attached to the specimen surface using an adhesive, and the signal acquisition threshold was set at 40 dB to minimize the influence of environmental noise and ensure the acquisition of valid AE signals. The PK6I sensors were equipped with built-in 26-dB low-noise preamplifiers for AE signal acquisition. The peak definition time (PDT), hit definition time (HDT), and hit lockout time (HLT) were set at 50, 150, and 300 μs, respectively.

## 3. Methodology

### 3.1. Acoustic Emission

AE technology is a nondestructive testing technique that utilizes elastic waves generated within materials to monitor damage evolution. Under SHPB impact loading, rock–concrete composite specimens are subjected to transient loading at high strain rates, resulting in complex and rapid damage evolution. The initial pores and microcracks within the specimens, together with defects in the rock–concrete ITZ, can generate local stress concentrations, thereby promoting rapid deformation and damage in localized regions. As the impact load increases, microcracks initiate and progressively propagate along the rock–concrete ITZ and within the concrete. As the impact load increases, microcracks initiate and progressively propagate along the rock–concrete interface and within the concrete. Once the elastic energy stored within the material reaches a critical level, crack initiation occurs, followed by progressive crack propagation until final fracture. The rapid release of elastic energy during this process generates elastic waves that propagate through the material and surrounding medium, thereby producing AE signals with distinct amplitude and frequency characteristics. These elastic waves are detected by AE sensors and subsequently converted into electrical signals. The resulting weak AE signals are then amplified by preamplifiers, recorded by the AE acquisition system, and processed to extract the corresponding characteristic parameters.

**Figure 3:**
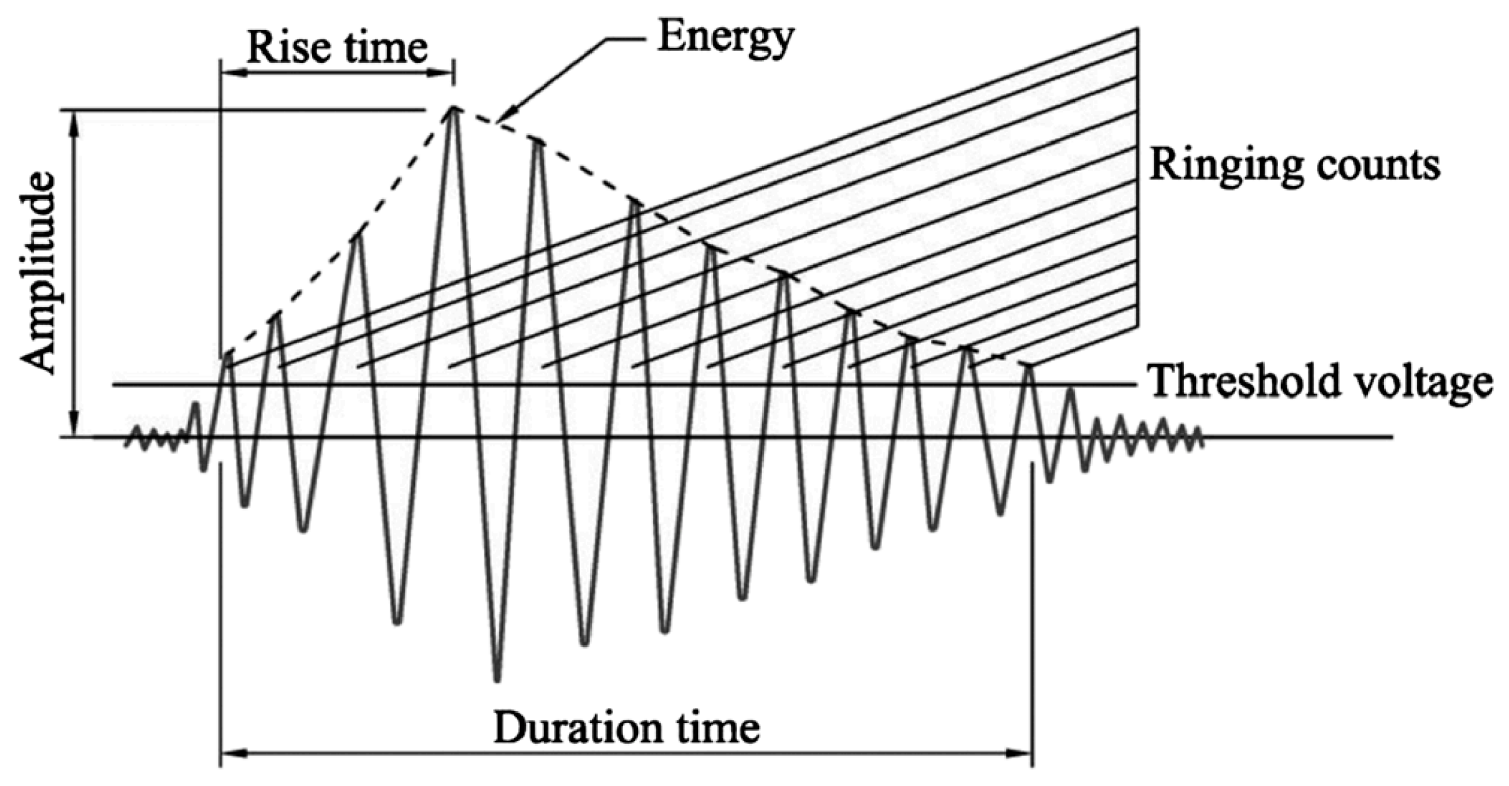
Definition of typical acoustic emission signal characteristic parameters.

AE signals contain information associated with material damage evolution, and their characteristic parameters can be used to characterize crack activity and failure processes in rock–concrete composite specimens. Common AE characteristic parameters include hit count, ring count, duration, peak amplitude, and energy. Among these parameters, hit count and ring count can indicate the degree of crack activity, whereas peak amplitude and energy can reflect variations in energy release during crack propagation. Therefore, analysis of these AE characteristic parameters can provide further insight into crack evolution and dynamic damage behavior in rock–concrete composite specimens under SHPB impact loading. Definitions of the commonly used AE characteristic parameters are presented in Figure 3.

### 3.2. Machine Learning-Based Damage Mode Classification

Methods based on AE characteristic parameters have been widely employed for crack-type identification in damage and failure studies of brittle materials, such as concrete and rock. The Japanese Concrete Institute standard JCMS-IIIB5706 [34] states that tensile and shear cracks can be distinguished based on the distributions of RA (RA = rise time/peak amplitude) and AF (AF = ring count/duration) values. However, the standard mainly provides a general approach for crack-type identification based on RA-AF parameters and does not specify a unified classification boundary that is applicable across different material properties and test conditions. In existing studies, the slope of the crack classification line is often determined empirically, and the resulting classification may be influenced by material properties, specimen dimensions, loading methods, and test environments [35]. Under SHPB impact loading at high strain rates, the rock–concrete ITZ is subjected to complex dynamic stresses, resulting in more complex crack initiation, propagation, and coalescence. Consequently, the corresponding AE feature data may exhibit nonlinear, overlapping, and multimodal distributions, thereby making conventional RA-AF criteria based on empirical boundaries inadequate for accurately characterizing damage-mode evolution during dynamic failure.

**Figure 4:**
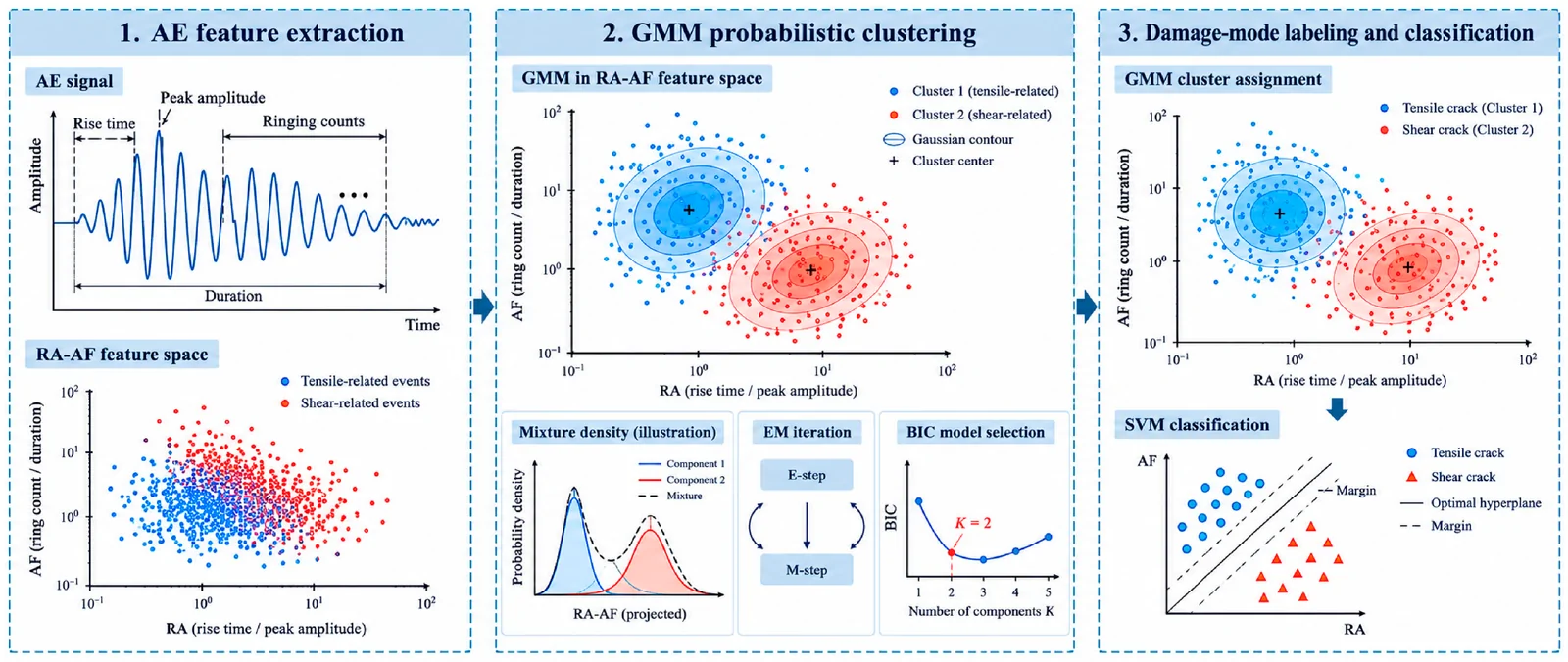
Schematic of the machine learning-based damage classification method.

To address these limitations and improve the objectivity of damage mode identification, a machine learning-based method combining the Gaussian mixture model (GMM) and support vector machine (SVM) was developed using the RA-AF characteristic parameters extracted from AE signals. The proposed method comprises two sequential stages. First, GMM is employed to characterize the underlying distribution of the AE feature data and to perform unsupervised clustering of the damage modes. Subsequently, an SVM classifier is trained using the GMM clustering results to automatically classify tensile and shear cracks. Compared with conventional approaches relying on empirically determined classification boundaries, the proposed method provides a data-driven framework for damage mode identification and provides a more quantitative basis for characterizing mixed-crack evolution and the corresponding damage mechanisms at rock–concrete interfaces under SHPB impact loading. A schematic illustration of the proposed machine learning-based damage classification method is shown in Figure 4.

The following section presents the relevant mathematical formulations and methodological procedures used in this study, including RA–AF feature representation, GMM-based clustering with a predefined number of Gaussian components, and subsequent SVM classification [36,37].

For each AE event, the corresponding RA and AF values were combined into a two-dimensional feature vector:

(1) $$X_{i}={\left[RA_{i},AF_{i}\right]}^{T}\in R^{2}\;(i=1,2,3\ldots N)$$

where *RA_i_* and *AF_i_* denote the RA and AF values of the *i*th AE event, respectively, and *N* is the total number of AE events.

Because crack propagation during SHPB impact is inherently random and complex, the AE feature data may exhibit multimodal distributions. Therefore, GMM was employed to model the probability distribution of the RA-AF feature data. GMM assumes that the observed samples are generated from a mixture of multiple Gaussian components, and its probability density function can be expressed as follows:

(2) $$p\left(X|\Theta \right)=\sum_{k=1}^{K}\omega_{k}\frac{1}{{\left(2\pi \right)}^{d/2}|\Sigma_{k}{|}^{1/2}}\exp\left[-\frac{1}{2}{\left(X-\mu_{k}\right)}^{T}\Sigma_{k}^{-1}\left(X-\mu_{k}\right)\right]$$

where *K* is the number of Gaussian components; *ω_k_* is the weight of the *k*th Gaussian component, satisfying $\sum_{k=1}^{K}\omega_{k}=1$; *μ_k_* and Σ*_k_* denote the mean vector and covariance matrix of the *k*th component, respectively; *d* is the feature dimension; and Θ represents the set of model parameters.

The GMM parameters were iteratively estimated and optimized using the expectation-maximization (EM) algorithm. In the E-step, the posterior probability of each sample belonging to each Gaussian component was calculated based on the current model parameters. In the M-step, the model parameters were re-estimated based on the calculated posterior probabilities. These two steps were repeated until the likelihood function converged to a maximum, yielding the final clustering result.

The Bayesian information criterion (BIC) is commonly used to evaluate GMMs with different numbers of Gaussian components and is expressed as follows:

*BIC*(*K*) = −2ln*L_K_* + *m_K_*ln*N* (3)

where *L_K_* is the maximum likelihood value of the model with *K* Gaussian components; *m_K_* is the number of model parameters; and *N* is the number of samples. In this study, the number of Gaussian components was fixed at *K* = 2 based on the physical objective of distinguishing two dominant damage modes, namely tensile and shear damage. This choice is consistent with our previous study [35], in which two Gaussian components were associated with these two damage modes. Accordingly, *K* = 2 was adopted for all four specimens to maintain a consistent two-mode classification framework.

In the present study, BIC was not used to optimize the number of Gaussian components for individual specimens. Accordingly, no specimen-specific BIC scores are reported.

Although GMM can reveal the underlying distribution of the AE feature space through unsupervised clustering, it does not directly provide an explicit decision boundary for subsequent damage mode classification. Therefore, SVM was further employed to develop a supervised classification model using the GMM clustering results. The SVM determines an optimal hyperplane that separates samples from different classes, and its decision function is expressed as follows:

*f*(*X*) = *w^T^X* + *b* (4)

where *w* is the normal vector of the hyperplane, representing the direction of the classification boundary, and *b* is the bias term, which adjusts the position of the classification boundary. A linear kernel was adopted, defined as $K\left(X_{i},X_{j}\right)=X_{i}^{T}X_{j}$, and the penalty parameter was set to *C* = 1. The corresponding soft-margin SVM optimization problem is expressed as follows:

(5) $$\begin{array}{c} \underset{w,b,\xi }{\min}\;\frac{1}{2}{\left\|w\right\|}^{2}+C\sum_{i=1}^{N}\xi_{i}\\ \mathrm{subject}\;\mathrm{to}\;y_{i}\left(w^{T}X_{i}+b\right)\geq 1-\xi_{i},\\ \;\;\;\;\;\xi_{i}\geq 0,\;\;i=1,2,\ldots ,N \end{array}$$

where *y_i_* ∈ {−1,+1} represents the class label derived from GMM clustering, and *ξ_i_* is the slack variable that allows classification violations. The penalty parameter *C* controls the trade-off between maximizing the classification margin and penalizing classification errors.

In this study, SVM was used to obtain an explicit linear decision boundary corresponding to the GMM-derived classes, rather than to evaluate independent out-of-sample predictive performance. Accordingly, all RA–AF feature vectors selected for damage-mode classification under the medium-impact-velocity condition were used to fit the SVM, without a separate training/test split. No independent test-set performance was therefore evaluated. The resulting GMM–SVM framework provides an explicit, data-driven boundary for distinguishing tensile- and shear-dominated damage based on AE RA–AF features, without relying solely on manually prescribed empirical criteria.

## 4. Results and Analysis

### 4.1. Failure Patterns

Table 3 presents the failure patterns of the specimens at different impact velocities. Under low-velocity impact conditions, the GC-1, GCF-1, SC-1, and SCF-1 specimens were able to maintain relatively intact original shapes. No obvious macroscopic cracks or fragmentation were observed on the specimen surfaces, indicating that the impact load had not yet reached a level sufficient to cause significant macroscopic damage. As the impact velocity further increased to the medium-velocity level, relatively obvious cracks began to appear on the surface of the GC-1 specimen, whereas the other three specimens remained essentially intact. This indicates that, with increasing impact load, pre-existing microcracks within the specimens gradually propagated and coalesced, eventually forming observable macroscopic cracks.

When the impact velocity reached the high-velocity level, clear differences in the degree of failure were observed among the different specimens. Both the GC-1 and SC-1 specimens underwent severe fragmentation, with the original specimen shapes largely lost and numerous block-like fragments formed. This indicates that the ordinary concrete composite specimens had experienced relatively extensive macroscopic failure under high-velocity impact. In comparison, although obvious cracks appeared in the GCF-1 specimen, its overall shape remained essentially intact, with no significant spalling observed. The SCF-1 specimen, however, began to exhibit fragmentation and localized spalling. These results indicate that the addition of fibers can, to some extent, delay crack propagation and the subsequent overall fragmentation caused by crack development, allowing the specimens to maintain better integrity under the same impact conditions.

Under ultra-high-velocity impact conditions, all four specimens underwent severe failure, with the original structural integrity largely lost and pronounced fragmentation and spalling observed. As the impact velocity further increased, the initiation, propagation, and coalescence of internal cracks within the specimens accelerated significantly. The continuous evolution of a large number of cracks ultimately led to complete fragmentation of the specimens. At this stage, although the bridging and restraining effects of the fibers could suppress crack propagation to some extent, they were insufficient to withstand the intense dynamic loading under ultra-high-velocity impact conditions. Consequently, the fiber-reinforced concrete composite specimens also exhibited severe fragmentation.

In summary, the failure patterns of the specimens exhibited a clear progressive evolution. The composite specimens consisting of ordinary concrete and fiber-reinforced concrete showed different failure evolution characteristics. Under high-velocity impact conditions, the GC-1 and SC-1 specimens had already undergone significant fragmentation, whereas the GCF-1 specimen retained good overall integrity, and the SCF-1 specimen exhibited only initial fragmentation and spalling. This indicates that the delaying effect of fiber reinforcement on crack propagation and failure development was more pronounced at medium to high impact velocities. This phenomenon demonstrates that the addition of fibers can improve the resistance of the concrete matrix to dynamic failure, thereby delaying the transition of the composite specimens from crack development to overall fragmentation.

### 4.2. Stress–Strain Response

**Figure 5:**
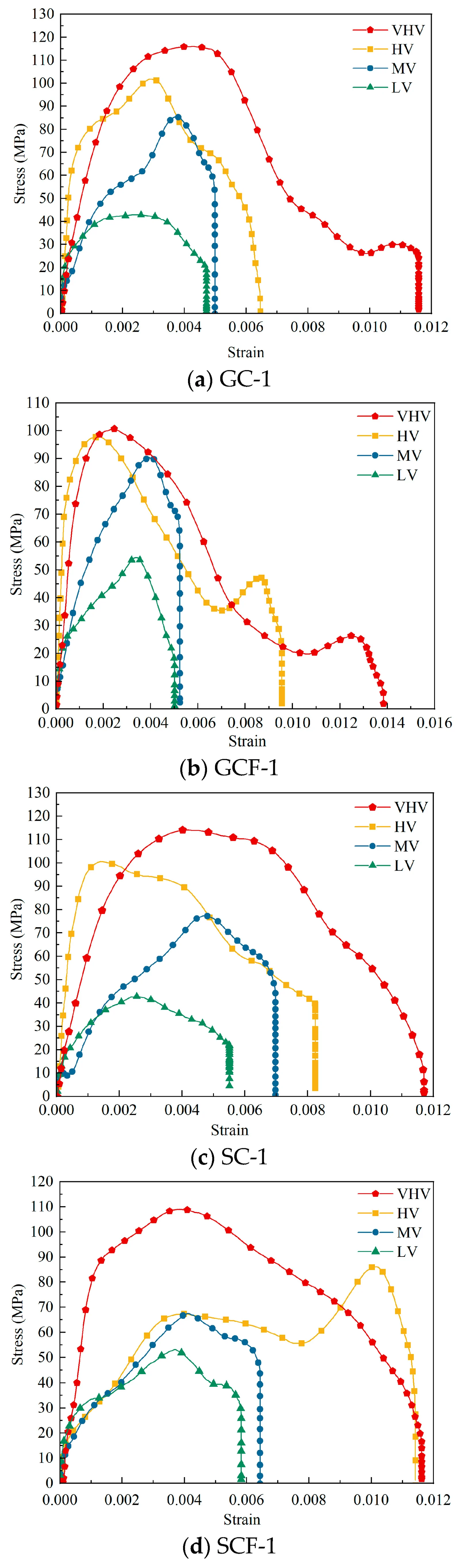
Stress–strain curves of rock–concrete composite specimens under various dynamic impact velocities.

The stress–strain curves characterize the deformation response of rock–concrete composite specimens under dynamic impact loading. Their peak stress, elastic response, and post-peak softening behavior provide key indicators of the dynamic load-bearing performance of the specimens. The dynamic stress–strain curves of all specimens under different impact velocities are shown in Figure 5, and the corresponding compressive strength parameters are listed in Table 4. Overall, the specimens exhibited similar progressive damage characteristics across the different impact velocities. The stress–strain response was divided into four stages: initial compaction, linear elastic growth, nonlinear damage, and post-peak softening. The curves generally showed an approximately linear increase toward the peak stress, followed by varying degrees of post-peak softening, which is characteristic of the dynamic compressive response of brittle materials.

During the initial loading stage, the slopes of the curves varied only slightly among the specimens, indicating relatively consistent initial elastic responses. This indicates that, under dynamic impact loading, the initial elastic deformation of the specimens is primarily governed by the overall stiffness of the composite and is relatively insensitive to variations in impact velocity. As the strain increased further, pre-existing pores, microcracks, and defects within the rock–concrete ITZ progressively closed, followed by a stable elastic deformation stage in which stress increased continuously with strain. As the loading approached the peak stress, microcracks within the specimens began to initiate and propagate rapidly, marking the transition to the nonlinear damage stage.

At comparable impact velocities, the peak stresses of the specimens differed markedly, with an overall increasing trend as the impact velocity increased, indicating a pronounced strain-rate strengthening effect in the rock–concrete specimens. This behavior is primarily attributed to the limited time available for crack initiation and propagation under high strain-rate conditions. Under such conditions, the development of internal defects is restricted, and a higher axial stress is therefore required to overcome the internal resistance of the material and induce complete failure.

In the post-peak stage, the specimens exhibited distinct softening behaviors. Both GC-1 and SC-1 showed a pronounced stress drop after reaching the peak stress, with GC-1 exhibiting a more rapid decline in stress. The steeper stress drop indicates a stronger tendency toward brittle failure in the granite-based composite specimen during the post-peak stage. This behavior is primarily attributed to the higher strength and elastic modulus of granite. These properties enhance the peak load-bearing capacity of the composite system while also enabling greater elastic energy storage. Once the cracks become interconnected, the stored energy is released rapidly, leading to a sharp drop in stress. In contrast, SC-1 exhibited a relatively gradual post-peak stress decline. The relatively high porosity and pre-existing defects within sandstone may facilitate progressive damage development, resulting in a more gradual failure process.

Fiber reinforcement further modified the post-peak response of the composite specimens, providing evidence of an enhanced toughening effect under dynamic loading. Compared with GC-1 and SC-1, GCF-1 and SCF-1 exhibited more gradual post-peak stress declines and retained greater residual load-bearing capacities. This behavior can be attributed to the fiber-bridging effect: fibers spanning developing cracks provide resistance to crack opening and delay crack coalescence. In addition, fiber–matrix debonding and pull-out may consume energy during crack propagation, allowing load transfer to be sustained as damage develops. These mechanisms prolong the post-peak strain-softening stage and mitigate the abrupt loss of load-bearing capacity associated with rapid crack propagation. The secondary peaks observed in GCF-1 under high- and ultra-high-impact-velocity conditions further suggest that fiber bridging and locally intact regions contributed to continued load bearing after the initial peak. Overall, fiber reinforcement improved post-peak load-bearing stability and impact damage tolerance, although the extent of this toughening effect depended on the rock type and impact velocity.

Notably, the granite-based composite specimens exhibited a pronounced double-peak stress response at high and ultra-high impact velocities. GC-1 exhibited a second peak only at ultra-high impact velocity, whereas GCF-1 exhibited a secondary peak at both high and ultra-high impact velocities. This response contrasts with the continuous softening observed in the sandstone-based composite specimens, suggesting that the granite-based specimens may undergo a staged failure process under high-strain-rate loading. The initial peak may correspond to crack development after the primary load-bearing structure reaches its limiting state, whereas the second peak may be associated with continued load-bearing by locally intact regions and residual load-bearing capacity provided by fiber bridging. In contrast, neither SC-1 nor SCF-1 exhibited an evident double-peak response, and both curves showed a relatively continuous post-peak decline, indicating a more progressive and uniform damage evolution in the sandstone-based composite systems.

Overall, impact velocity, rock type, and fiber reinforcement collectively influenced the dynamic stress–strain response of the rock–concrete specimens. Impact velocity primarily governed the peak stress, reflecting the strain-rate strengthening effect, whereas the mechanical properties of the rock and fiber reinforcement mainly affected post-peak damage evolution and residual load-bearing behavior. Owing to its relatively high strength and brittle nature, granite was more prone to staged failure at high strain rates. Sandstone, with its more developed internal defect structure, exhibited a more gradual damage evolution. The dynamic deformation stability of the composite specimens was enhanced by fiber reinforcement through modifications to interfacial restraint and crack propagation patterns.

### 4.3. Dynamic Compressive Strength

**Figure 6:**
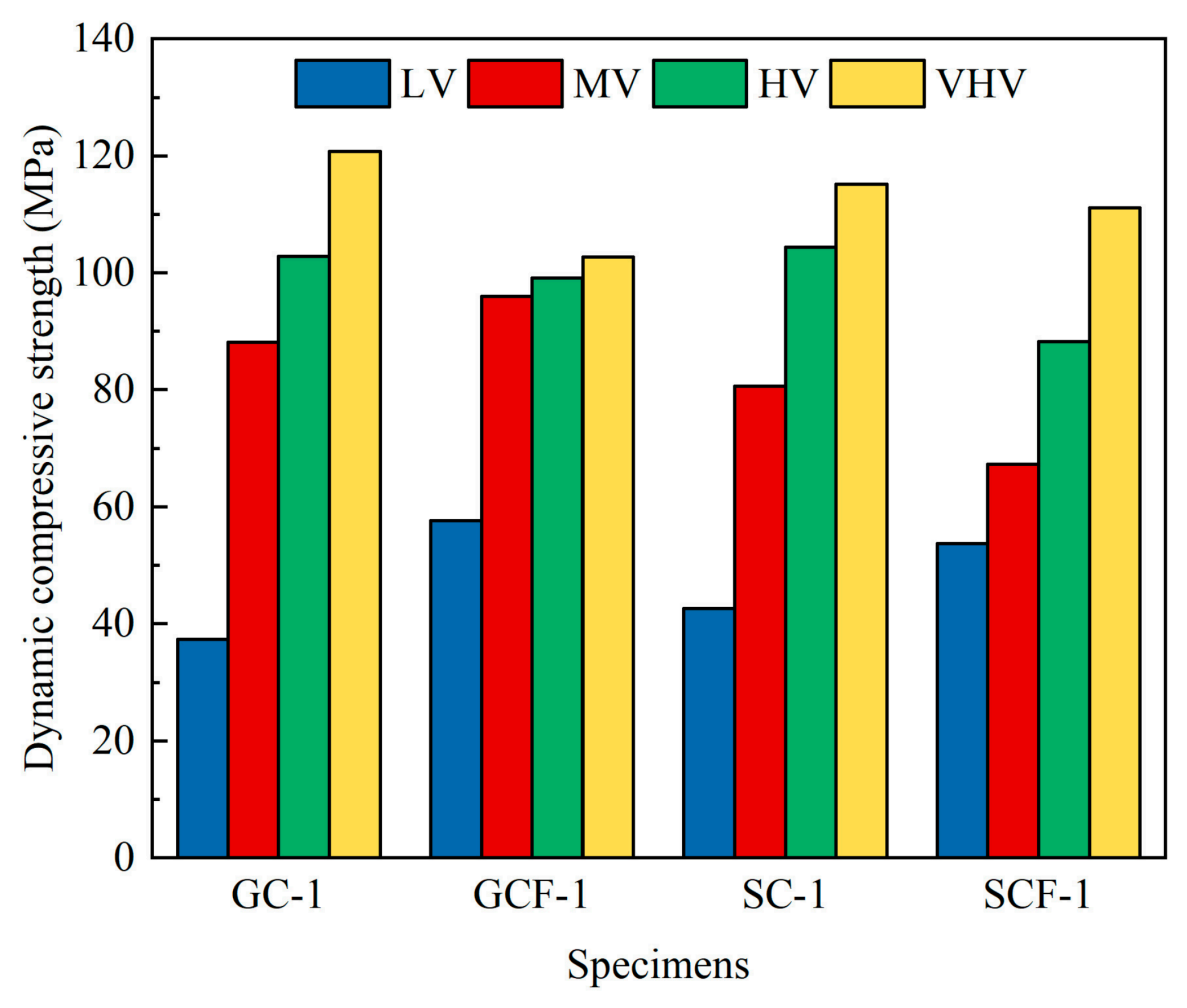
Dynamic compressive strength of specimens under various impact velocities.

Dynamic compressive strength reflects the load-bearing capacity of rock–concrete composite specimens under high-strain-rate impact loading and serves as an important indicator of the dynamic mechanical performance of the composite systems. The dynamic compressive strengths of the specimens at different impact velocities are shown in Figure 6. Overall, the dynamic compressive strength of all specimen types increased progressively with increasing impact velocity, indicating a pronounced strain-rate strengthening effect. This behavior suggests that, as the loading rate increases, the initiation and propagation of internal cracks are increasingly constrained by the limited time available for damage evolution. Consequently, damage evolution may lag behind the increase in external loading, allowing the composite systems to sustain higher impact stresses before failure.

For the GC-1 specimens, the dynamic compressive strength increased from 37.36 MPa at low impact velocity to 120.75 MPa at ultra-high impact velocity. The strength reached 88.15 MPa and 102.77 MPa at medium and high impact velocities, respectively, indicating a pronounced strain-rate strengthening response in the granite–concrete specimens at higher impact velocities. The dynamic compressive strength of GCF-1 increased from 57.65 MPa to 102.73 MPa, representing an increase of 78.22%. However, its strength increased more gradually with impact velocity, suggesting that fiber reinforcement enhanced the initial load-bearing capacity of the specimens while reducing the sensitivity of dynamic compressive strength to variations in impact velocity.

The sandstone-based composite specimens also exhibited a pronounced strain-rate strengthening effect. The dynamic compressive strength of SC-1 increased from 42.59 MPa to 115.15 MPa, while that of SCF-1 increased from 53.72 MPa to 111.14 MPa. For SC-1, the dynamic compressive strength reached 104.38 MPa and 115.15 MPa at high and ultra-high impact velocities, respectively, and these values were comparable to those of the granite-based composite specimens. This indicates that, under high-strain-rate loading, impact-induced strengthening can substantially diminish the influence of differences in the intrinsic rock strength on the macroscopic load-bearing capacity of the composite system.

The variation in strength across different impact velocities reveals clear differences among the specimens, particularly at low impact velocities. For example, the dynamic compressive strengths of GCF-1 and SCF-1 were 57.65 MPa and 53.72 MPa, respectively, under low-impact-velocity conditions, and were both notably higher than those of GC-1 and SC-1. This indicates that fiber reinforcement enhanced the initial load-bearing capacity of the composite specimens. This improvement is primarily attributed to the restraint provided by the fibers within the concrete matrix, which increases resistance to damage during crack initiation and influences stress transfer across the rock–concrete ITZ.

As the impact velocity increased further, the influence of rock type on the dynamic compressive strength gradually decreased. Under high and ultra-high impact velocities, the differences in strength among the specimen groups became less pronounced, indicating that inertial and dynamic confinement effects played an increasingly important role in governing the load-bearing capacity of the composite systems at high strain rates. During high-strain-rate loading, crack propagation is increasingly constrained, resulting in a more complex internal stress state. Consequently, the dynamic load-bearing capacity is primarily governed by the loading rate and interfacial stress transfer.

The effect of fiber reinforcement on dynamic compressive strength was more pronounced at low and medium impact velocities, whereas its contribution to peak strength became less evident at ultra-high impact velocity. For example, at ultra-high impact velocity, the dynamic compressive strengths of GC-1 and GCF-1 were 120.75 MPa and 102.73 MPa, respectively, while those of SC-1 and SCF-1 were 115.15 MPa and 111.14 MPa, respectively. The enhancement in peak strength provided by fiber reinforcement decreased at ultra-high impact, which may be attributed to the increasing dominance of dynamic loading effects at high strain rates, thereby reducing the relative contribution of fiber reinforcement to the peak load-bearing capacity of the specimens. Under these conditions, fiber reinforcement mainly enhances resistance to crack propagation and thereby improves post-peak load-bearing stability.

Overall, impact velocity, rock type, and fiber reinforcement all influenced the dynamic compressive behavior of the rock–concrete specimens. Impact velocity played the dominant role in the strain-rate strengthening effect and was the primary factor contributing to the increase in dynamic compressive strength. Fiber reinforcement enhanced resistance to damage by increasing interfacial restraint and modifying crack propagation. Rock type mainly influenced the initial load-bearing capacity and dynamic failure mode of the composite systems. Within the scope of this study, the effects of high-strain-rate loading and interfacial reinforcement became increasingly more pronounced than those of the intrinsic rock properties in determining the dynamic mechanical response of the composite specimens.

### 4.4. Dynamic Compressive Strength Increase Factor

**Figure 7:**
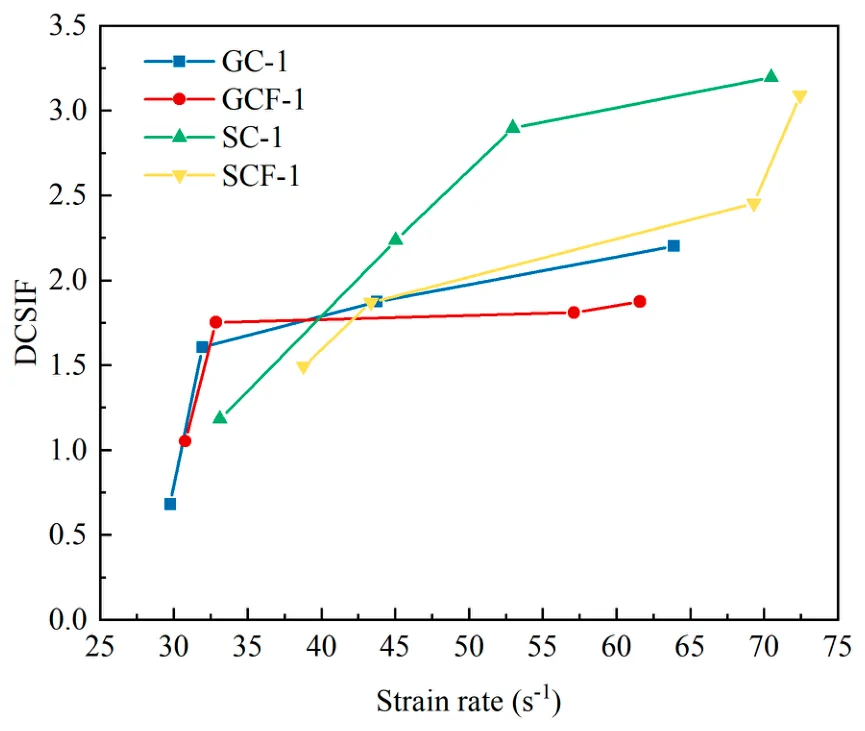
Variation in DCSIF with strain rate for specimens.

The dynamic compressive strength increase factor (DCSIF) is defined as the ratio of the dynamic compressive strength to the static compressive strength. It is an important indicator of the relative increase in material strength under dynamic impact loading. It is calculated as follows:

(6) $$\mathrm{DCSIF}=\frac{f_{d}}{f_{s}}$$

where *f_d_* denotes the dynamic compressive strength and *f_s_* denotes the static compressive strength. Compared with using dynamic compressive strength alone to evaluate the impact resistance of materials, DCSIF accounts for differences in initial strength and provides a more representative basis for comparing the relative dynamic strengthening responses of rock–concrete composite specimens. The variation in DCSIF for the specimens at different impact velocities is shown in Figure 7.

As shown in Figure 7, the DCSIF of all rock–concrete composite specimens increased with increasing strain rate, indicating a pronounced strain-rate strengthening effect. This trend is consistent with the increase in dynamic compressive strength with impact velocity, indicating that higher strain rates are associated with a greater relative increase in strength under dynamic loading. With increasing impact velocity, the damage evolution within the specimens was increasingly influenced by high-strain-rate loading, resulting in a more pronounced strengthening response. Consequently, the dynamic compressive strength increased relative to the corresponding static compressive strength, leading to an increase in DCSIF. Although all specimens exhibited strain-rate strengthening, the magnitude of the increase varied considerably, indicating that rock type and fiber reinforcement also influenced their dynamic strengthening behavior.

For the granite-based composite specimens, both GC-1 and GCF-1 exhibited increasing DCSIF values with increasing strain rate, indicating similar strain-rate strengthening trends. The DCSIF of GC-1 increased from 0.68 under low-impact-velocity conditions to 2.20 under ultra-high-impact-velocity conditions, whereas that of GCF-1 increased from 1.05 to 1.87. A comparison of the two specimens shows that GCF-1 exhibited higher DCSIF values than GC-1 under low- and medium-impact-velocity conditions, whereas its DCSIF values were lower under high- and ultra-high-impact-velocity conditions. These results indicate that the influence of fiber reinforcement on the dynamic strengthening behavior of granite-based composite specimens varied with impact velocity, resulting in different strengthening characteristics under different impact conditions.

For the sandstone-based composite specimens, SC-1 and SCF-1 also exhibited increasing DCSIF values with increasing strain rate. The DCSIF of SC-1 increased from 1.18 under low-impact-velocity conditions to 3.19 under ultra-high-impact-velocity conditions, whereas that of SCF-1 increased from 1.49 to 3.08. Under low-impact-velocity conditions, SCF-1 exhibited a higher DCSIF than SC-1; however, under medium-, high-, and ultra-high-impact-velocity conditions, the DCSIF of SCF-1 was lower than that of SC-1. These results indicate that, for sandstone-based composite specimens, fiber reinforcement was associated with a higher DCSIF under low-impact-velocity conditions, whereas lower DCSIF values were observed under medium- and higher-impact-velocity conditions. Taken together with the results for the granite-based specimens, these findings demonstrate that the influence of fiber reinforcement on DCSIF depended on impact velocity and varied with rock type.

A comparison of specimens with different rock types indicates that the sandstone-based specimens generally exhibited higher DCSIF values than the granite-based specimens at corresponding impact-velocity levels. The larger DCSIF values and fitting slopes of the sandstone-based specimens indicate a more pronounced dynamic strengthening response and greater sensitivity to strain rate. Meanwhile, the comparisons between ordinary-concrete and fiber-reinforced specimens demonstrate that the influence of fiber reinforcement depends on both rock type and impact velocity.

**Figure 8:**
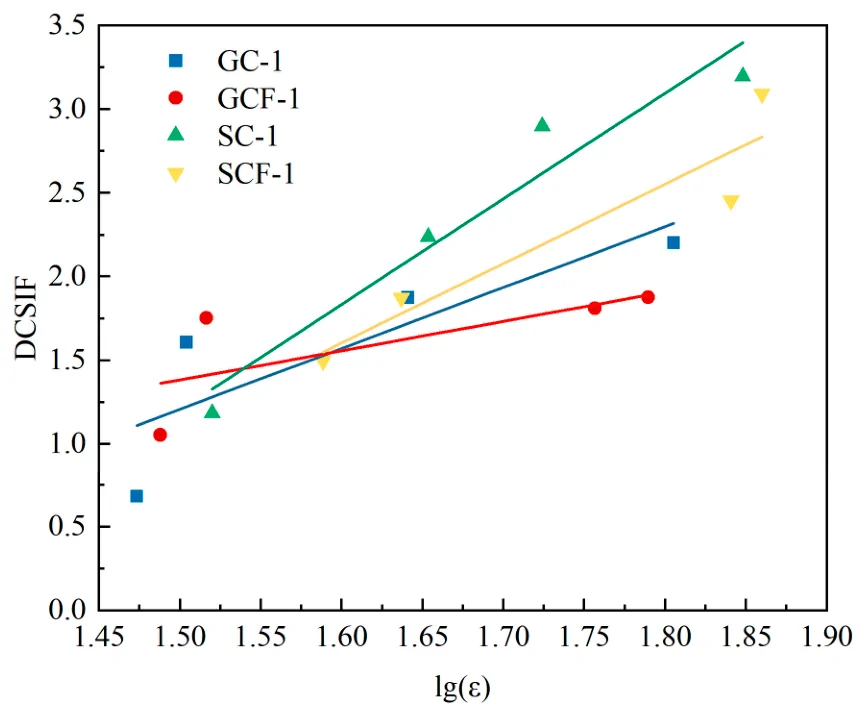
Relationship between DCSIF and logarithmic strain rate.

To further examine the quantitative relationship between DCSIF and strain rate, a fitting analysis was performed by fitting DCSIF against the logarithmic strain rate, lg($\dot{\varepsilon }$), as shown in Figure 8. The fitting relationship can be expressed as follows:

(7) $$\mathrm{DCSIF}=a\mathrm{lg}\left(\dot{\varepsilon }\right)+b$$

where *a* is the slope of the fitted line, representing the sensitivity of DCSIF to changes in strain rate, and *b* is the fitting intercept. The DCSIF fitting results are presented in Table 5.

The fitting results indicate that DCSIF exhibits an approximately linear relationship with lg($\dot{\varepsilon }$) for all specimens, suggesting that the logarithmic strain rate provides a reasonable representation of the strain-rate strengthening behavior of the rock–concrete specimens.

The fitted relationships exhibited clear differences among the specimens. The sandstone-based specimens showed larger fitting slopes than the granite-based specimens, indicating a greater increase in DCSIF with logarithmic strain rate. Specifically, the fitting slopes were 6.31358 and 4.74208 for SC-1 and SCF-1, respectively, compared with 3.63591 and 1.75186 for GC-1 and GCF-1. These results indicate that the DCSIF of the sandstone-based specimens was more sensitive to changes in strain rate, whereas the granite-based specimens exhibited a more gradual strain-rate strengthening trend. The coefficients of determination (R^2^) were also higher for the sandstone-based specimens (0.939 for SC-1 and 0.889 for SCF-1) than for the granite-based specimens (0.711 for GC-1 and 0.518 for GCF-1), indicating a stronger linear relationship between DCSIF and logarithmic strain rate in the sandstone-based specimens.

A comparison of the fitting parameters further shows that the fiber-reinforced specimens had smaller fitting slopes than their corresponding ordinary-concrete specimens for both rock types. This indicates that fiber reinforcement was associated with a lower rate of increase in DCSIF with logarithmic strain rate. The differences in fitting slopes were more pronounced for the granite-based specimens, with the slope decreasing from 3.63591 for GC-1 to 1.75186 for GCF-1, while the corresponding decrease for the sandstone-based specimens was from 6.31358 for SC-1 to 4.74208 for SCF-1. In addition, the ordinary-concrete specimens exhibited slightly higher coefficients of determination than their corresponding fiber-reinforced specimens, indicating a somewhat stronger linear fit between DCSIF and logarithmic strain rate. These fitting results complement the specimen-level comparisons presented above and further demonstrate that fiber reinforcement modified the strain-rate strengthening characteristics of the composite specimens.

Overall, the DCSIF of all rock–concrete composite specimens increased with increasing strain rate, while the sandstone-based specimens generally exhibited a more pronounced dynamic strengthening response than the granite-based specimens. The influence of fiber reinforcement varied with rock type and impact velocity. For both rock types, the fiber-reinforced specimens exhibited higher DCSIF values under low-impact-velocity conditions, whereas lower values were observed under high- and ultra-high-impact-velocity conditions. At medium impact velocity, GCF-1 exhibited a higher DCSIF than GC-1, while SCF-1 exhibited a lower DCSIF than SC-1. Combined with the dynamic stress–strain responses, these findings indicate that fiber reinforcement modified the strain-rate strengthening behavior and failure process of the composite specimens, with the resulting DCSIF response depending on rock type and impact conditions.

### 4.5. Energy Absorption Capacity

**Figure 9:**
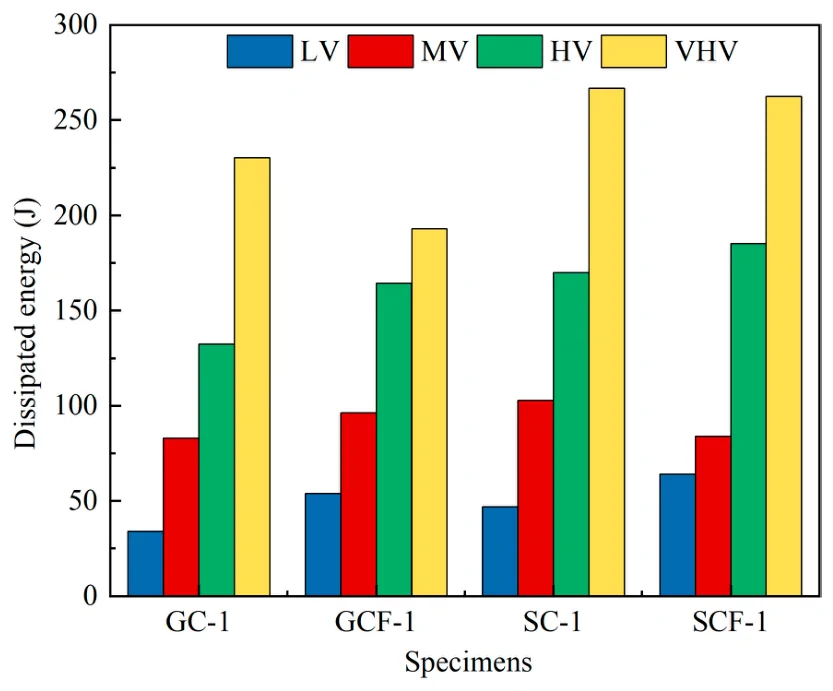
Dissipated energy of different specimens under various impact velocities.

**Figure 10:**
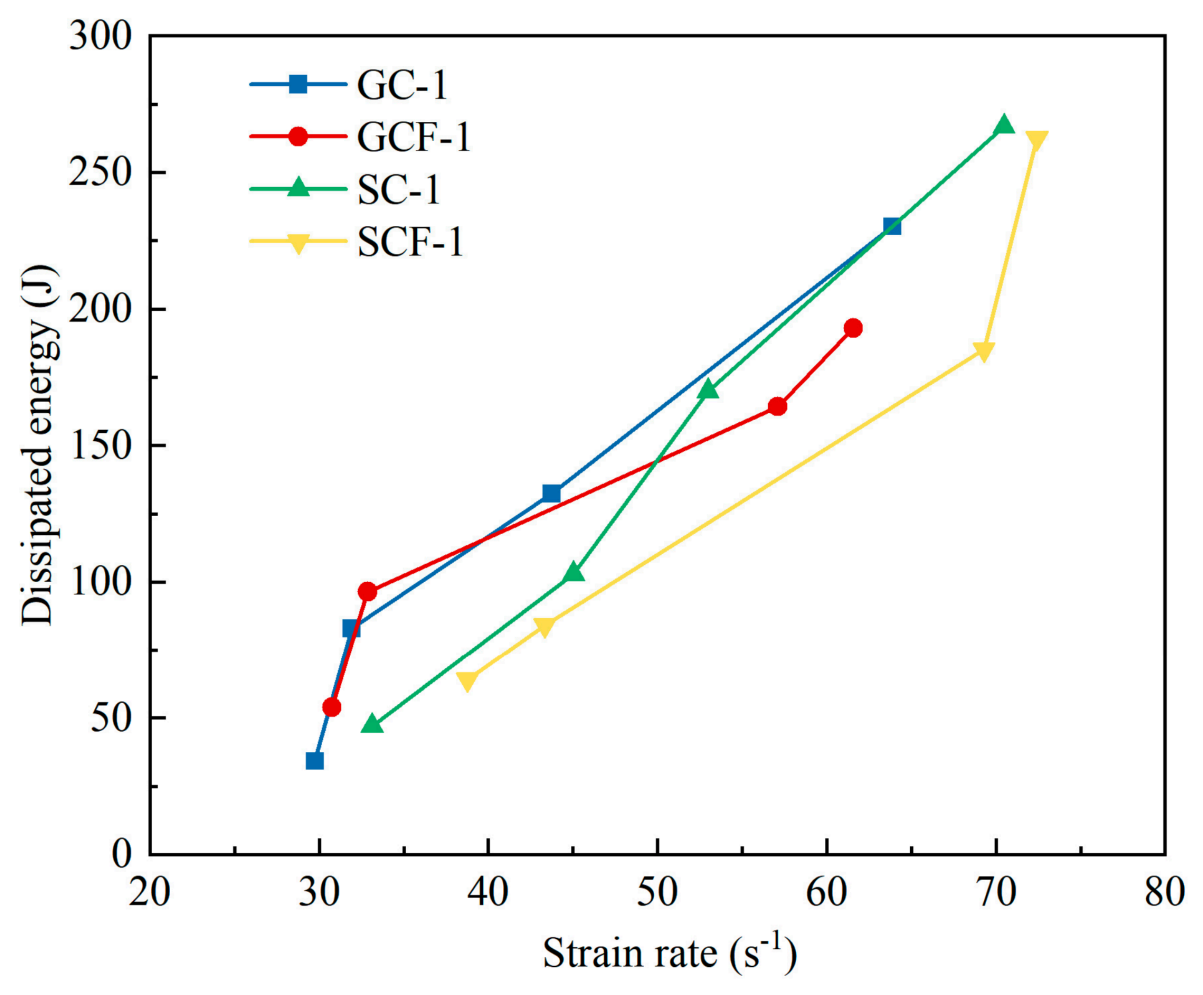
Dissipated energy–strain rate curves of different specimens.

Dissipated energy reflects the energy consumed by irreversible processes such as crack propagation, damage within the rock–concrete ITZ, and material fragmentation during dynamic impact loading of the rock–concrete specimens. It therefore provides an important basis for assessing the damage state of the specimens. The dissipated energy of the specimens at different impact velocities is shown in Figure 9, and the corresponding dissipated energy–strain rate relationships are presented in Figure 10. The dissipated energy of all rock–concrete specimens increased with increasing impact velocity, while the corresponding dissipated energy–strain rate relationships exhibited an overall positive correlation. This indicates that high-strain-rate impact loading promotes crack initiation, propagation, and coalescence within the specimens, thereby resulting in greater energy dissipation during failure.

For GC-1, the dissipated energy increased from 34.19 J at low impact velocity to 230.19 J at ultra-high impact velocity. The dissipated energy–strain rate relationship exhibited a clear increasing trend, particularly from high to ultra-high impact velocity, during which dissipated energy increased sharply from 132.33 J to 230.19 J. This indicates that crack propagation in the granite–concrete specimens became more pronounced with increasing strain rate, resulting in greater energy dissipation during failure. The dissipated energy of GCF-1 increased from 53.92 J to 192.98 J, showing an overall trend similar to that of GC-1, although the variation differed across the different impact velocity levels. Notably, the dissipated energies of GCF-1 at low, medium, and high impact velocities were 53.92, 96.37, and 164.22 J, respectively, all of which were higher than the corresponding values for GC-1. However, at ultra-high impact velocity, the dissipated energy of GCF-1 was 192.98 J, which was lower than the 230.19 J measured for GC-1. This indicates that the effect of fiber reinforcement on dissipated energy varied across the different impact velocity levels. The fiber bridging effect can restrict rapid crack propagation and modify crack propagation patterns, while impact energy is dissipated through crack friction, fiber pull-out, and interfacial damage.

The sandstone-based rock–concrete specimens exhibited a distinct evolution of dissipated energy. The dissipated energy of SC-1 increased from 46.95 J to 266.81 J and remained higher than that of GC-1 across all impact velocities. The dissipated energy–strain rate relationship for SC-1 exhibited a more rapid increase at high strain rates, indicating that the relatively well-developed pore structure and pre-existing defects in sandstone may have facilitated crack initiation and propagation, thereby increasing energy dissipation during dynamic failure. In contrast, the dissipated energy of SCF-1 increased from 64.12 J to 262.44 J. Similar to SC-1, SCF-1 exhibited an increasing energy dissipation trend with strain rate, although its dissipated energy varied across the different impact velocity levels. At low impact velocity, the dissipated energy was 64.12 J, which was higher than the 46.95 J measured for SC-1. At medium impact velocity, it increased to 84.04 J, although this value was lower than the 102.86 J measured for SC-1. At high impact velocity, it increased again to 185.14 J, exceeding the 169.85 J measured for SC-1. At ultra-high impact velocity, it reached 262.44 J, which was slightly lower than the 266.81 J measured for SC-1. These results indicate that fiber reinforcement affects crack propagation and energy dissipation, with its influence varying with impact velocity.

A comparison of the dissipated energy–strain rate relationships among specimens with different rock types indicates that both granite-based and sandstone-based specimens exhibited clear strain-rate sensitivity, although their energy dissipation patterns differed. At low and medium impact velocities, the difference in dissipated energy between the two rock types remained relatively small. As the impact velocity increased, however, the sandstone-based specimens exhibited higher levels of dissipated energy. At ultra-high impact velocity, the dissipated energies of SC-1 and SCF-1 reached 266.81 J and 262.44 J, respectively, compared with 230.19 J and 192.98 J for GC-1 and GCF-1, respectively. This may indicate that the sandstone-based specimens developed more complex crack propagation paths under high strain rates, thereby enabling greater impact-energy absorption and dissipation.

The toughening effect of fiber reinforcement should be interpreted in conjunction with both the post-peak stress–strain response and the dissipated energy. The measured dissipated energy did not increase consistently with fiber addition at every impact velocity. Instead, fiber reinforcement modified the progression of damage and the pathways of energy dissipation. Fiber bridging restrained crack opening and delayed crack coalescence, while fiber pull-out, crack-surface friction, and interfacial damage provided additional mechanisms for energy consumption. Consequently, the beneficial effect of fiber reinforcement was reflected not simply in the magnitude of dissipated energy, but also in the more gradual post-peak stress decline, the retention of residual load-bearing capacity, and the delayed development of severe fragmentation. These observations indicate that fiber reinforcement enhanced the resistance of the composite specimens to unstable crack propagation, although its effect on total dissipated energy remained dependent on impact velocity and rock type.

Overall, the dissipated energy of the rock–concrete specimens was primarily governed by impact velocity. As the strain rate increased, crack propagation and damage accumulation became more pronounced, leading to a continuous increase in dissipated energy. Rock type affected the crack propagation patterns within the specimens, with the sandstone-based specimens exhibiting greater energy dissipation at high strain rates. Fiber reinforcement modified the energy dissipation process by regulating crack propagation, thereby contributing to a more stable failure process.

### 4.6. Acoustic Emission Hit Count Analysis

**Figure 11:**
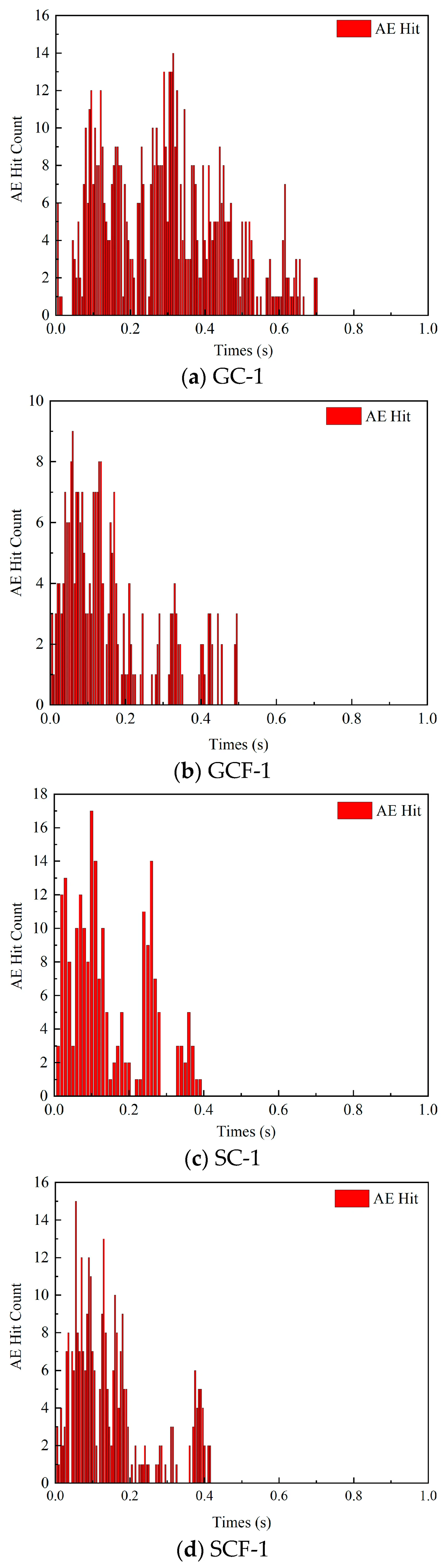
AE Hit Count-Time Curves of Specimens Under MV Impact Conditions.

AE hit count reflects the initiation and propagation of internal microcracks and the associated damage-release process, making it an important parameter for characterizing the dynamic failure behavior of rock–concrete composite specimens. Since transient impact waves generated during high-speed loading may interfere with AE sensor measurements, the medium-impact-velocity condition was selected for AE testing to ensure stable signal acquisition. The AE hit count-time distributions of the specimens are shown in Figure 11. Compared with static or quasi-static loading, SHPB impact loading occurs over a much shorter time interval, with damage evolution within the specimens occurring predominantly during the transient impact process. Accordingly, the AE signals exhibit a pronounced concentration of AE activity.

As shown in Figure 11, all specimens exhibited a pronounced transient concentration of AE hit activity during the early stage of impact. Most hit events occurred within 0–0.5 s, after which the AE activity gradually declined. This indicates that most of the detectable internal damage developed during the early stage of impact loading. As cracks propagated rapidly and formed the primary failure zone, the energy available for subsequent damage release gradually decreased, resulting in fewer AE events. Differences in the distribution of AE hit counts among the specimens indicate the effects of rock type and fiber reinforcement on dynamic damage evolution.

For the granite-based composite specimens, both GC-1 and GCF-1 exhibited relatively concentrated AE activity during the early impact stage. GC-1 exhibited a higher number of AE hit events, with pronounced fluctuations during the early and intermediate stages of impact. This indicates that the relatively high strength and integrity of granite allow the specimen to accumulate considerable elastic energy under impact loading, followed by a concentrated release of damage associated with rapid crack propagation. In comparison, GCF-1 exhibited a lower overall AE hit count, with most events concentrated during the initial impact stage. This suggests that fiber reinforcement restrained rapid crack propagation and reduced the concentration of damage-related AE events, resulting in a more stable failure process.

The sandstone-based composite specimens, SC-1 and SCF-1, exhibited AE activity patterns that were distinct from those of the granite-based specimens. SC-1 exhibited a relatively high rate of AE hit events during the early impact stage, followed by a rapid decline in AE activity. This indicates that the pre-existing defects and relatively high porosity of sandstone may facilitate microcrack propagation, resulting in more rapid damage release. Although SCF-1 also exhibited a relatively high level of AE activity during the initial impact stage, its overall AE activity was more widely distributed, with a measurable number of AE events occurring during the later stage. This suggests that fiber bridging delayed crack coalescence and shifted the damage evolution from concentrated failure toward more progressive crack propagation.

A comparison of specimens with different rock types indicates that the granite-based specimens exhibited more pronounced instantaneous damage release, whereas AE activity in the sandstone-based specimens was more widely distributed. This reflects differences in damage evolution associated with the distinct internal structures and mechanical properties of the two rock types. The relatively high strength and dense structure of granite may lead to more abrupt crack propagation, whereas the greater abundance of internal defects in sandstone may facilitate progressive crack growth and associated energy release. In addition, the AE hit counts of the fiber-reinforced concrete specimens were generally lower than those of the corresponding specimens with ordinary concrete, indicating that fiber reinforcement may enhance damage transfer at the rock–concrete interface and reduce the concentration of damage release associated with rapid crack propagation.

### 4.7. Machine Learning-Based Damage Mode Classification Results

**Figure 12:**
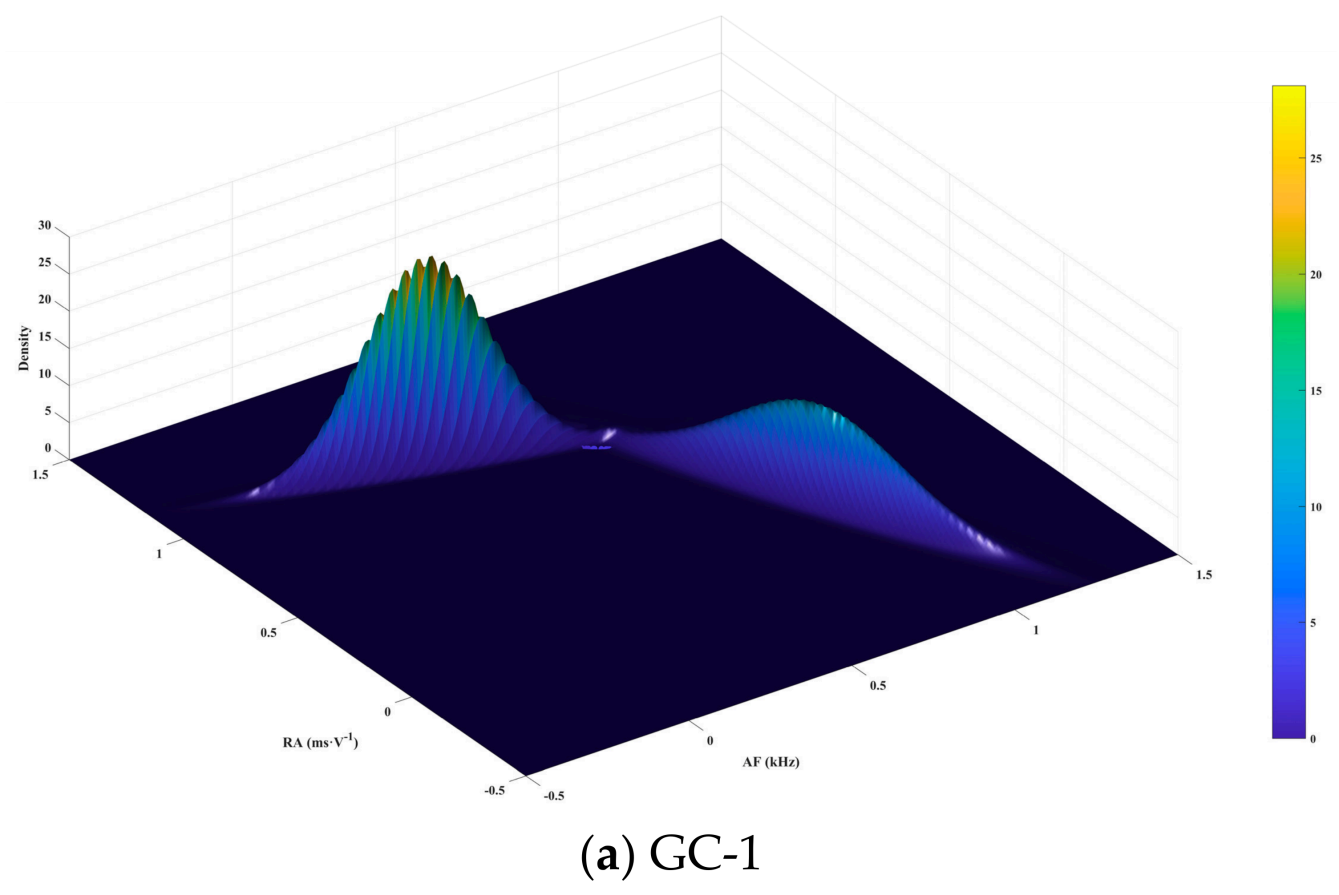

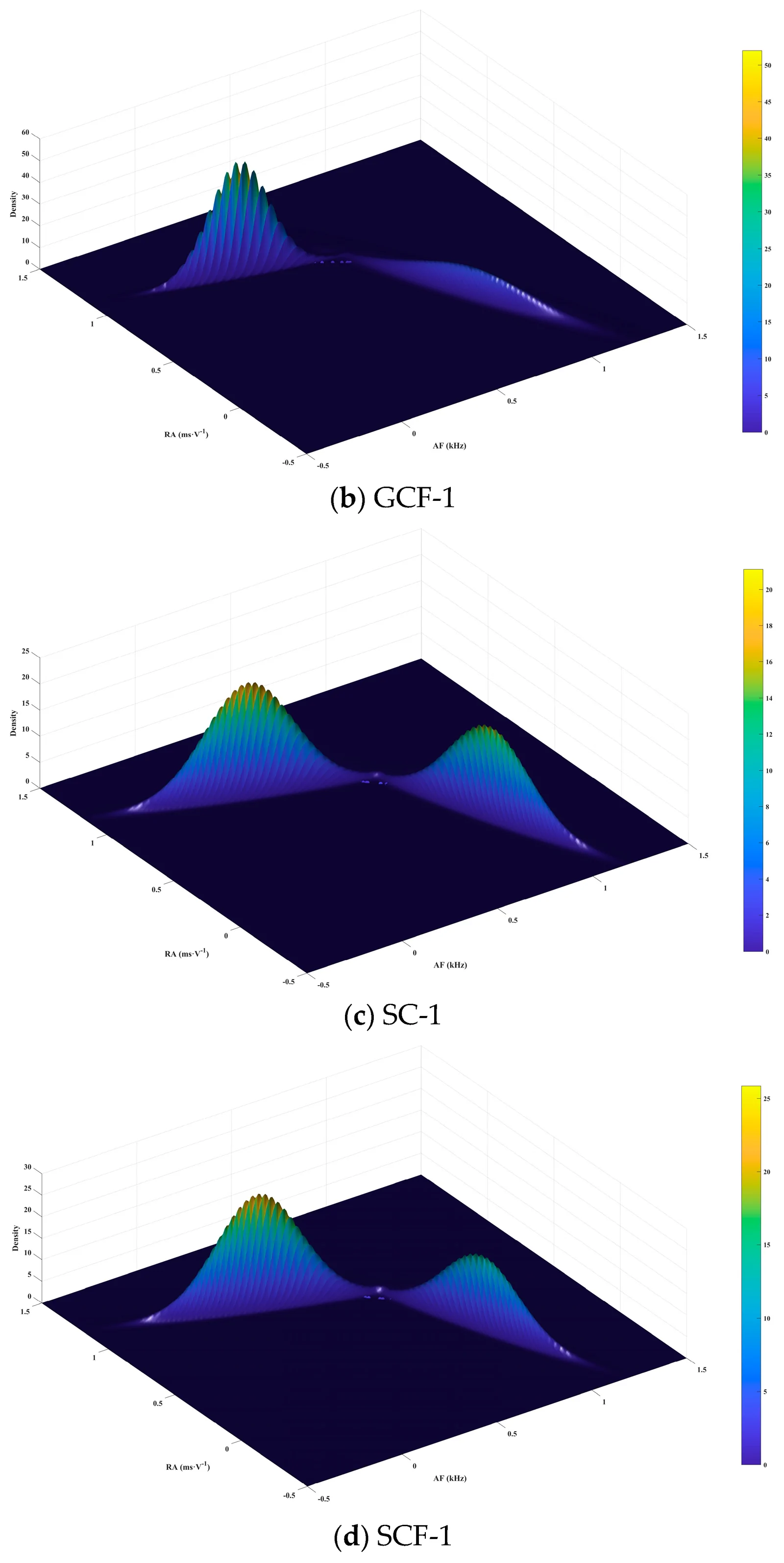
Normalized Probability Density Distribution of RA-AF Clustering Based on GMM.

The distribution of AE characteristic parameters provides valuable information for distinguishing damage modes under impact loading. During dynamic impact, complex local damage evolution is induced by stress-wave propagation, stress concentration within the rock–concrete ITZ, and material heterogeneity within the specimens. The relative proportions of tensile and shear damage therefore indicate the dominant failure mechanisms of the rock–concrete composite specimens. Conventional RA-AF analysis generally classifies individual AE events using an empirically defined boundary; however, the resulting classification may be affected by material type, specimen configuration, and loading conditions. To reduce the uncertainty associated with manually defined classification boundaries, the proposed GMM–SVM method was applied to the RA-AF feature data to perform data-driven damage mode classification. Because transient effects during high-speed impact may affect the stability of AE measurements, the AE tests considered in this analysis were conducted at a medium impact velocity. The GMM-derived normalized probability density distributions of the RA-AF data are shown in Figure 12.

**Figure 13:**
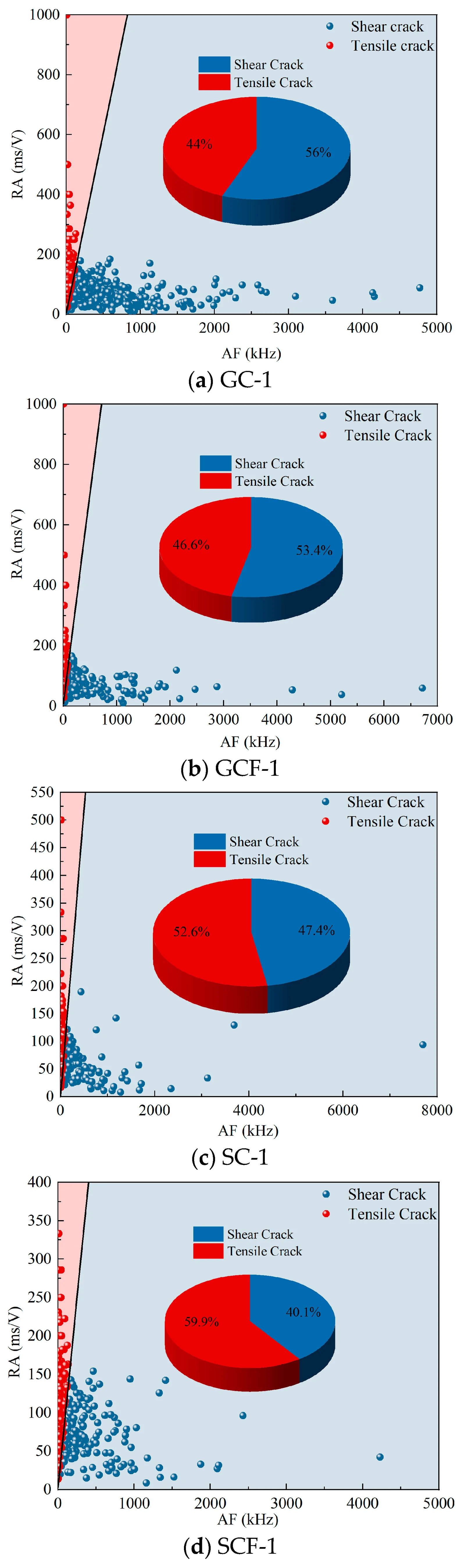
Classification results of damage modes.

It should be noted that, in the normalized RA-AF probability density plots, the height of a probability peak represents the concentration of AE events within a specific region of the feature space and reflects the similarity and compactness of the corresponding signal characteristics. It does not directly indicate the proportion or dominance of a particular damage mode during the actual failure process. Therefore, to quantitatively characterize the damage modes of the specimens, the AE events were further classified as tensile or shear damage using the GMM–SVM method, and the corresponding proportions were calculated for each specimen. The GMM–SVM-based damage classification results derived from the AE RA-AF features are presented in Figure 13.

As shown in Figure 13, the rock–concrete composite specimens exhibited distinct differences in damage modes between the two rock types. The shear damage ratios of GC-1 and GCF-1 were 56.0% and 53.4%, respectively, both exceeding their corresponding tensile damage ratios. This indicates that shear damage was the dominant damage mode in the granite-based composite specimens under dynamic impact loading. Granite is characterized by relatively high strength and good structural integrity. Once cracks initiate within the rock, their propagation is strongly constrained, which may promote relative sliding along localized damage planes and induce local shear failure in stress-concentrated regions. In addition, the mismatch in mechanical properties between rock and concrete can generate relatively high shear stresses within and around the rock–concrete ITZ during dynamic loading, thereby contributing to more pronounced shear-dominated damage evolution. Accordingly, the granite-based composite specimens exhibited predominantly shear damage.

In contrast, the sandstone-based composite specimens exhibited a distinct damage-mode distribution. The tensile damage ratios of SC-1 and SCF-1 reached 52.6% and 59.9%, respectively, both exceeding the corresponding shear damage ratios. This indicates that tensile damage was the dominant failure mode in the sandstone-based composite specimens. Sandstone generally contains more pores and inherent defects, which can promote localized tensile stress concentrations under dynamic impact loading. These weak regions may facilitate microcrack initiation and opening, followed by progressive crack propagation. The presence of internal defects also reduces the resistance to crack propagation, allowing damage to develop more readily in tensile mode. Consequently, compared with the granite-based specimens, the sandstone-based specimens exhibited a higher proportion of tensile damage.

The results further demonstrate that rock type had a pronounced effect on the damage-mode distribution of the rock–concrete composite specimens. Shear damage was dominant in the granite-based specimens, whereas tensile damage was predominant in the sandstone-based specimens. This difference suggests that the inherent structural characteristics of the rock influence stress transfer and damage propagation during dynamic impact loading. Within the composite system, the rock layer affects not only the overall load-bearing response but also the local stress state near the rock–concrete interface, thereby contributing to differences in damage-mode distribution.

Fiber reinforcement also affected the damage-mode distribution. Compared with the corresponding specimens containing ordinary concrete, the tensile damage ratios of GCF-1 and SCF-1 were 46.6% and 59.9%, respectively, with a more pronounced increase observed in SCF-1. This result indicates that fiber reinforcement modifies the damage propagation pattern within the concrete layer. Through fiber bridging, the fibers can restrain rapid crack propagation and increase resistance to crack growth, causing local damage to develop progressively rather than through rapid instability. During this process, some shear-sliding damage may be suppressed, whereas the proportion of tensile-related AE events associated with crack opening and propagation may increase under fiber restraint. Therefore, fiber reinforcement does not simply reduce the extent of damage; rather, it modifies the relative distribution and evolution of different damage modes during dynamic failure.

Overall, the GMM–SVM-based RA-AF classification results revealed clear differences in the dynamic damage-mode distribution of rock–concrete composite specimens with different rock types and reinforcement conditions. Shear damage was predominant in the granite-based specimens, whereas tensile damage dominated in the sandstone-based specimens. Fiber reinforcement increased the proportion of tensile damage, particularly in the sandstone-based specimens, and modified the subsequent damage evolution pattern. These findings demonstrate that the proposed GMM–SVM learning approach can quantitatively distinguish damage modes based on AE RA-AF features and provide further insight into the effects of rock type and fiber reinforcement on damage evolution in rock–concrete composite specimens under impact loading.

## 5. Conclusions

(1) All specimens exhibited a pronounced strain-rate strengthening effect, with their dynamic stress–strain responses successively progressing through the initial compaction, elastic, nonlinear damage, and post-peak softening stages. Peak dynamic stress generally increased with increasing impact velocity. Granite-based specimens exhibited a distinct double-peak response at high and ultra-high impact velocities, whereas sandstone-based specimens exhibited more continuous post-peak softening, indicating distinct damage evolution characteristics between the two rock types.

(2) The dynamic compressive strength of all specimens increased with increasing impact velocity, demonstrating the strong effect of loading rate on dynamic load-bearing capacity. At low impact velocities, fiber-reinforced specimens generally exhibited higher dynamic compressive strength than those with ordinary concrete; however, the contribution of fiber reinforcement to peak strength became less pronounced with increasing impact velocity.

(3) The DCSIF of all specimens increased approximately linearly with increasing strain rate. Sandstone-based specimens exhibited greater strain-rate sensitivity, with generally higher fitting slopes and coefficients of determination than the granite-based specimens. The influence of fiber reinforcement on DCSIF varied with rock type and impact velocity. For both rock types, fiber-reinforced specimens exhibited higher DCSIF values under low-impact-velocity conditions, whereas lower values were observed under high- and ultra-high-impact-velocity conditions. At medium impact velocity, GCF-1 exhibited a higher DCSIF than GC-1, while SCF-1 exhibited a lower DCSIF than SC-1.

(4) Dissipated energy increased with both impact velocity and strain rate, indicating greater energy dissipation during dynamic failure. Sandstone-based specimens generally exhibited greater dissipated energy than granite-based specimens, while fiber reinforcement reduced dissipated energy for a given rock type, suggesting that fiber reinforcement mainly modified crack propagation and energy-release characteristics rather than simply increasing energy dissipation.

(5) The GMM–SVM machine-learning method based on AE RA–AF features effectively distinguished between tensile and shear damage modes. To ensure signal stability, damage-mode classification was conducted exclusively under medium-impact-velocity conditions; therefore, the identified damage-mode distributions should be interpreted within this specific loading condition. Granite-based specimens were dominated by shear damage, whereas tensile damage predominated in sandstone-based specimens. Fiber reinforcement increased the proportion of tensile damage for both rock types, indicating that rock type and fiber reinforcement had distinct effects on the distribution and evolution of dynamic damage modes.

## Figures and Tables

**Table 1:** Mix Proportion Design of Concrete.

Mix	Cement (kg/m^3^)	Coarse Aggregate (kg/m^3^)	Fine Aggregate (kg/m^3^)	Fiber (kg/m^3^)	Water (kg/m^3^)	Water Reducer (%)
Plain concrete	475	789	964	0	190	1.0
Fiber concrete	475	775	948	10	190	1.0

**Table 2:** Basic Properties of Rock and Concrete.

Performance Parameter	Plain Concrete	Fiber Concrete	Granite	Sandstone
Compressive strength (MPa)	52.5	52.3	130.3	67.6
Elasticity modulus (GPa)	36.4	35.8	48.9	19.0

**Table 3:** Failure patterns of specimens.

No.	LV	MV	HV	VHV
GC-1	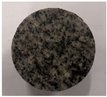	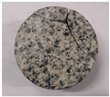	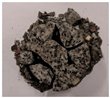	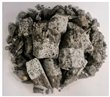
GCF-1	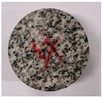	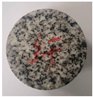	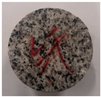	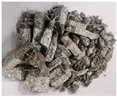
SC-1	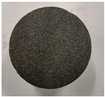	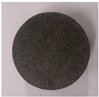	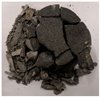	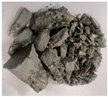
SCF-1	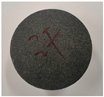	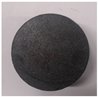	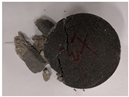	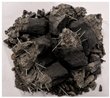

**Table 4:** Compressive performance parameters of the tested specimens under different impact conditions.

No.	Impact Velocity (m/s)	Strain Rate (s^−1^)	Static Compressive Strength (MPa)	Dynamic Compressive Strength (MPa)	DCSIF	Dissipated Energy (J)
GC-1	3.180	29.74	54.84	37.35	0.68	34.194
	5.125	31.92		88.14	1.60	82.982
	6.263	43.74		102.77	1.87	132.327
	7.570	63.88		120.75	2.20	230.192
GCF-1	3.579	30.76	54.75	57.65	1.05	53.918
	5.193	32.85		95.99	1.75	96.369
	6.404	57.11		99.08	1.80	164.218
	7.531	61.59		102.73	1.87	192.980
SC-1	3.561	33.12	36.03	42.58	1.18	46.948
	5.084	45.03		80.55	2.23	102.861
	6.317	52.98		104.38	2.89	169.847
	7.982	70.47		115.15	3.19	266.809
SCF-1	3.665	38.77	35.97	53.71	1.49	64.119
	4.816	43.35		67.28	1.87	84.040
	6.575	69.30		88.22	2.45	185.135
	7.471	72.42		111.14	3.08	262.440

**Table 5:** Fitted DCSIF curve expression.

No.	DCSIF–lg($\dot{\varepsilon }$) Fitting	*R* ^2^
GC-1	DCSIF = 3.63591$\mathrm{lg}\left(\dot{\varepsilon }\right)$ − 4.248	0.711
GCF-1	DCSIF = 1.75186$\mathrm{lg}\left(\dot{\varepsilon }\right)$ − 1.24683	0.518
SC-1	DCSIF = 6.31358$\mathrm{lg}\left(\dot{\varepsilon }\right)$ − 8.26982	0.939
SCF-1	DCSIF = 4.74208$\mathrm{lg}\left(\dot{\varepsilon }\right)$ − 5.98449	0.889

## Data Availability

The original contributions presented in this study are included in the article. Further inquiries can be directed to the corresponding authors.
